# Supplementary material for: Effectiveness of a Mobile App in Reducing Therapeutic Turnaround Time and Facilitating Communication between Caregivers in a Pediatric Emergency Department: A Randomized Controlled Pilot Trial
Source: J Pers Med. 2022 Mar 9;12(3):428. doi: 10.3390/jpm12030428 (PMC8948631; doi:10.3390/jpm12030428)
Supplement: Supplementary file 1 [file jpm-12-00428-s001.zip › jpm-1617873-supplementary.pdf]

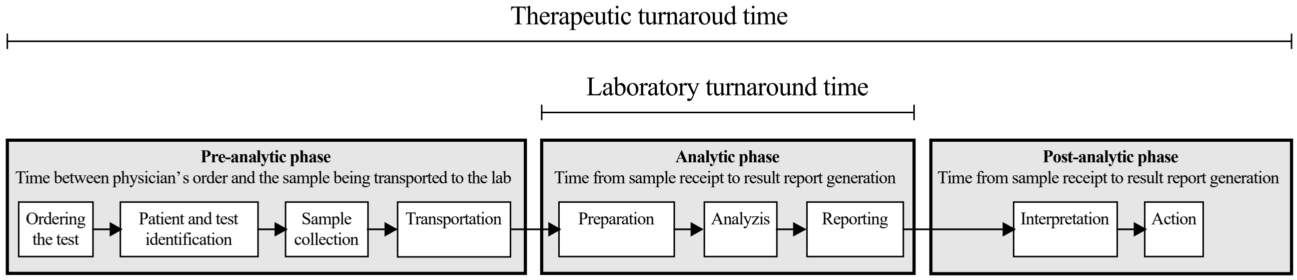

Supplementary Figure S1. The therapeutic turnaround time. The therapeutic turnaround time can be divided schematically into three phases, i.e., two extra-laboratory phases (pre- and post-analytical) and a third, analytical, intra-laboratory phase. The pre-analytical phase refers to the time from the physician's order of tests to the receipt of samples at the laboratory. The analytical phase (including the laboratory turnaround time) extends from the latter to the production and availability of a report containing the results, generally provided today in a computerized way. The post-analytical phase consists of the physician receiving the report and interpreting it to make a decision. According to Lundberg [63], these three phases can also be further subdivided into nine stages (white boxes). Adapted from [64].

63. Lundberg GD. Acting on significant laboratory results. *JAMA* 1981;245(17):1762-1763.
64. Pati HP, Singh G. Turnaround Time (TAT): Difference in Concept for Laboratory and Clinician. *Indian J Hematol Blood Transfus* 2014;30(2):81-84.
